# Accurate measurement of chest compression depth using impulse-radio ultra-wideband sensor on a mattress

**DOI:** 10.1371/journal.pone.0183971

**Published:** 2017-08-30

**Authors:** Byung Gyu Yu, Je Hyeok Oh, Yeomyung Kim, Tae Wook Kim

**Affiliations:** 1 School of Electrical and Electronic Engineering, Yonsei University, Seoul, Republic of Korea; 2 Department of Emergency Medicine, College of Medicine, Chung-Ang University, Seoul, Republic of Korea; Azienda Ospedaliero Universitaria Careggi, ITALY

## Abstract

**Objective:**

We developed a new chest compression depth (CCD) measuring technology using radar and impulse-radio ultra-wideband (IR-UWB) sensor. This study was performed to determine its accuracy on a soft surface.

**Methods:**

Four trials, trial 1: chest compressions on the floor using an accelerometer device; trial 2: chest compressions on the floor using an IR-UWB sensor; trial 3: chest compressions on a foam mattress using an accelerometer device; trial 4: chest compressions on a foam mattress using an IR-UWB sensor, were performed in a random order. In all the trials, a cardiopulmonary resuscitation provider delivered 50 uninterrupted chest compressions to a manikin.

**Results:**

The CCD measured by the manikin and the device were as follows: 57.42 ± 2.23 and 53.92 ± 2.92 mm, respectively in trial 1 (*p* < 0.001); 56.29 ± 1.96 and 54.16 ± 3.90 mm, respectively in trial 2 (*p* < 0.001); 55.61 ± 1.57 and 103.48 ± 10.48 mm, respectively in trial 3 (*p* < 0.001); 57.14 ± 3.99 and 55.51 ± 3.39 mm, respectively in trial 4 (*p* = 0.012). The gaps between the CCD measured by the manikin and the devices (accelerometer device vs. IR-UWB sensor) on the floor were not different (3.50 ± 2.08 mm vs. 3.15 ± 2.27 mm, respectively, *p* = 0.136). However, the gaps were significantly different on the foam mattress (48.53 ± 5.65 mm vs. 4.10 ± 2.47 mm, *p* < 0.001).

**Conclusion:**

The IR-UWB sensor could measure the CCD accurately both on the floor and on the foam mattress.

## Introduction

A deeper chest compression is associated with improved survival outcomes in cardiac arrest patients [1, 2]. Although several feedback devices have been developed to achieve adequate chest compression depth (CCD), the effects of the feedback devices were not examined in a clinical setting [3–5]. In addition, the feedback devices using accelerometer technique could overestimate the CCD when the chest compression was performed on soft surfaces, such as foam or inflatable mattresses [6]. Recently, several technologies were reported to overcome this drawback [7–9]. One such technology, the TrueCPR (Physio-Control, Redmond, Washington, USA), which uses a three-dimensional magnetic field, was produced as a commercially available device. The TrueCPR has been proved to measure the CCD accurately on soft surfaces and to improve the CCD when using feedback functions [10, 11]. However, there are some limitations in using the TrueCPR device in a clinical setting, because a large-sized back pad (74 mm × 266 mm ×100 mm) should be placed under the patient’s thorax to measure the CCD accurately on soft surfaces. The size of the chest pad is also not small (35 mm × 225.6 mm × 83 mm), and it has a hard surface. Although another technique, which uses a flexible pressure sensor, could overcome the drawbacks of TrueCPR, it has not been produced as a commercially available device [12].

**Fig 1:**
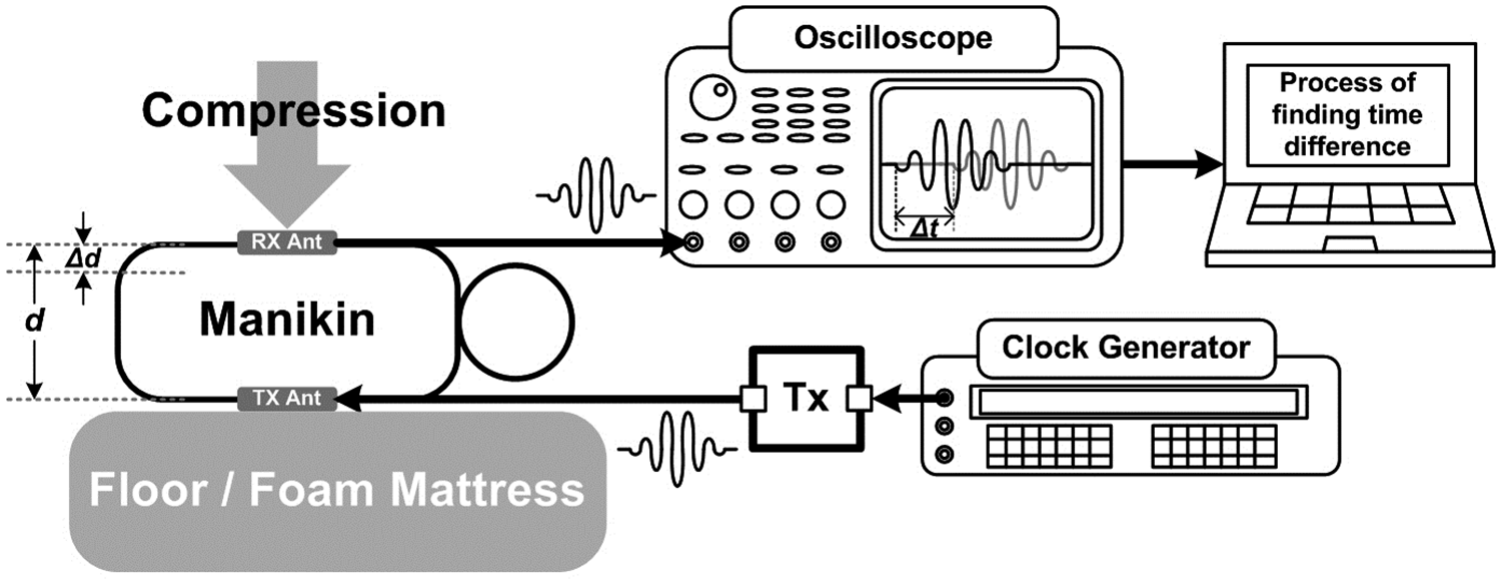
Chest compression depth measurement using impulse-radio ultra-wideband sensor. Tx: transmitter; Tx Ant.: transmitter antenna; Rx Ant.: receiver antenna; *Δd*: chest compression depth; *Δt*: the time difference between the received signal before chest compression and that according to the location of the receiver which changes in real time during chest compressions.

We developed a new CCD measuring technology, which utilizes an impulse-radio ultra-wideband (IR-UWB) sensor (Fig 1) [13]. This study was performed to determine its accuracy on hard and soft surfaces, using a manikin.

## Materials and methods

### Design

Four trials, trial 1: chest compressions on the floor using an accelerometer device; trial 2: chest compressions on the floor using an IR-UWB sensor; trial 3: chest compressions on a foam mattress using an accelerometer device; trial 4: chest compressions on a foam mattress using an IR-UWB sensor, were performed in a random order. In all the trials, a cardiopulmonary resuscitation (CPR) provider delivered 50 uninterrupted chest compressions to a manikin. The four trials were conducted in numerical order.

### The principles of chest-compression depth measurement using IR-UWB sensor

**Fig 2:**
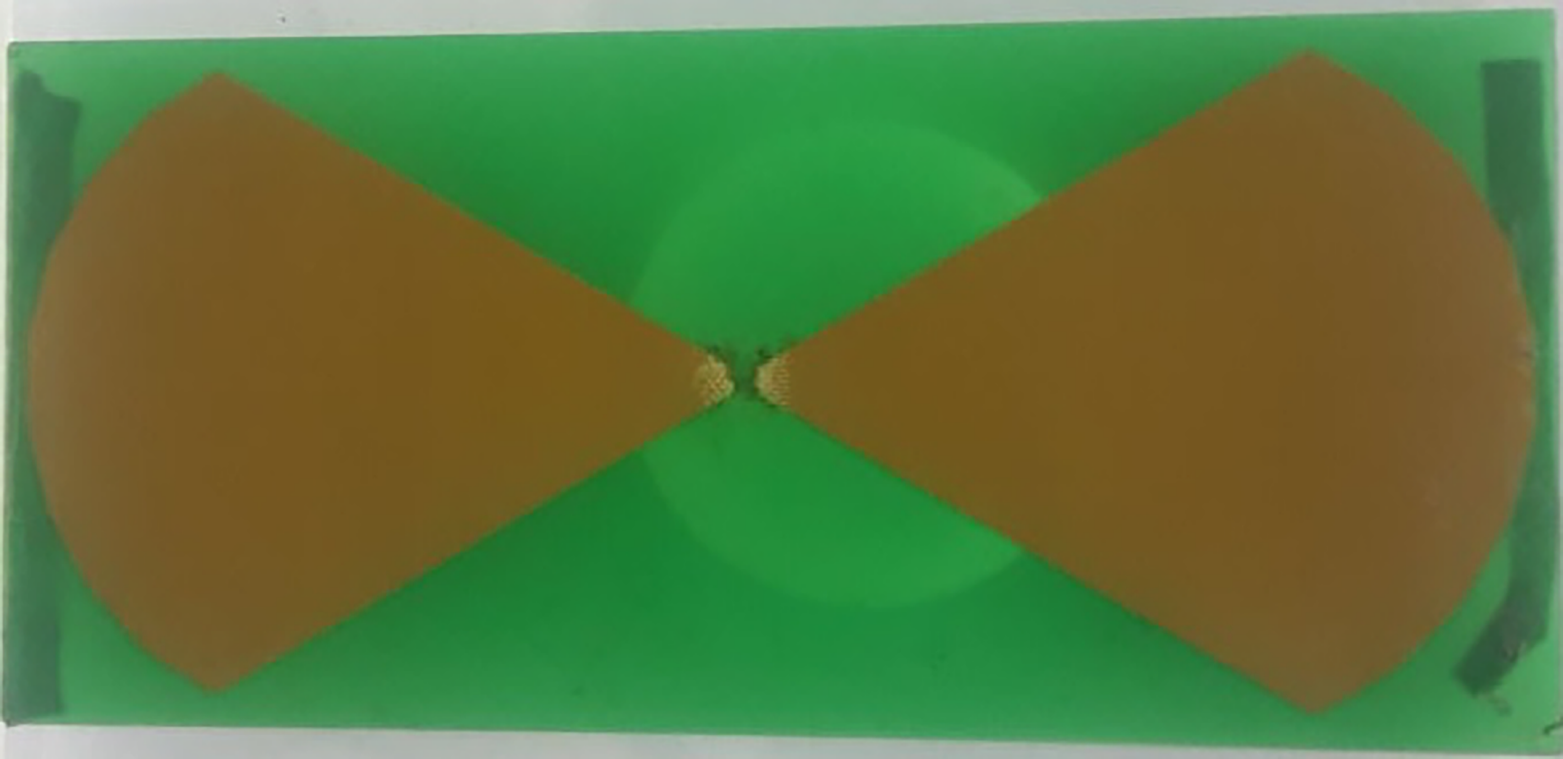
Patch antenna printed circuit board (22 cm × 10 cm).

The transmitter antenna is located under the thorax of the manikin and the receiver antenna is located on the anterior chest wall of the manikin (Fig 2). The distance between the two antennas (*d*) is determined using the following equation:

d=c×t

The constant *c* refers to the speed of light (2.9979 × 10^8^ m/s) and *t* refers to the time difference measured at the receiver. The value of *d* changes in real time during the chest compressions. By using a time-difference-of-arrival technique, the CCD (*Δd*) can be calculated by the following equation:

Δd=c×Δt

**Fig 3:**
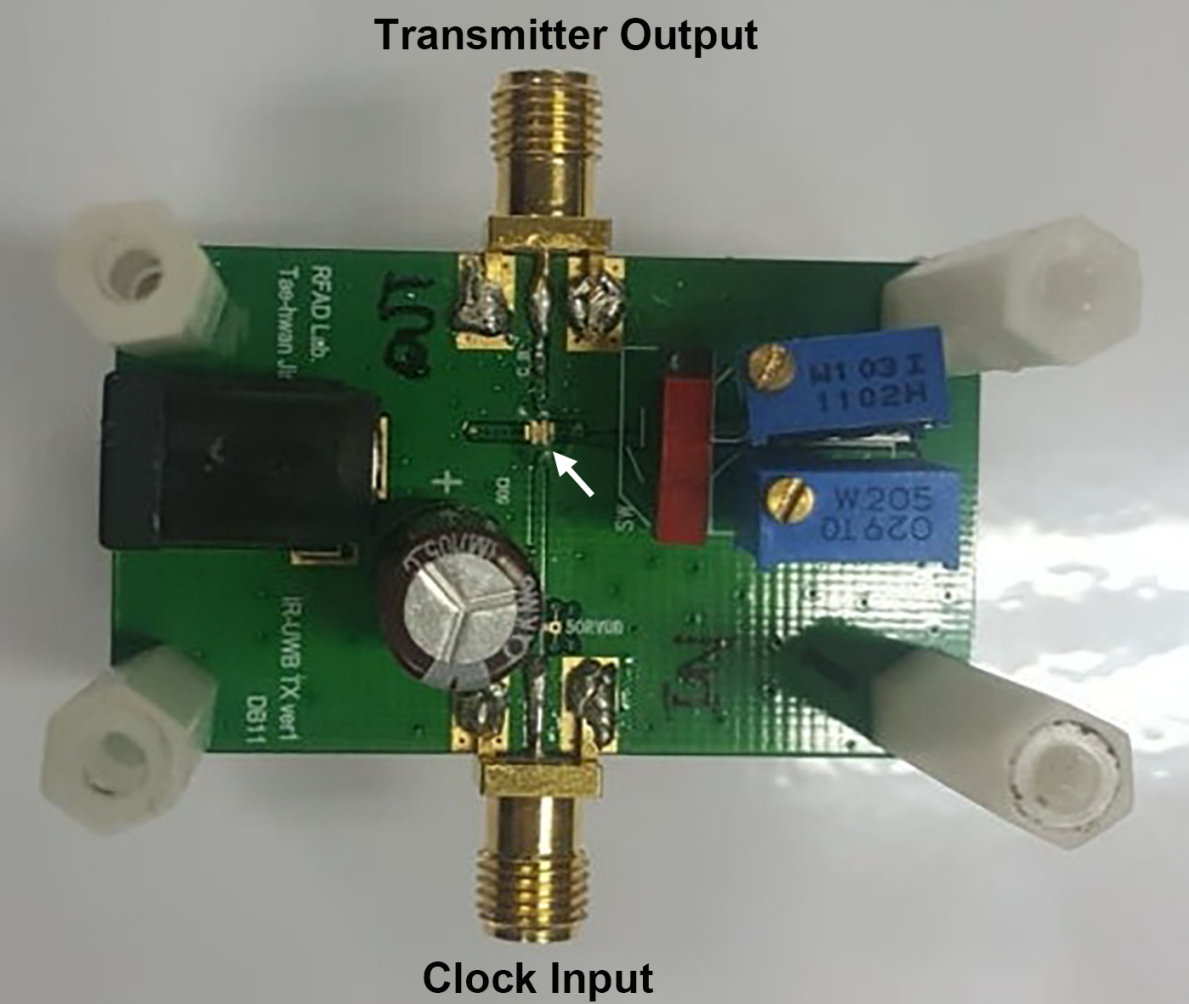
Transmitter system-on-chip printed circuit board (5 cm × 3 cm). White arrow: transmitter system-on-chip designed in 65nm CMOS process (1 mm × 0.4 mm).

Here, *Δt* refers to the time difference between the received signal before chest compression and that according to the location of the receiver, which changes in real time during chest compressions. The signals generated from the transmitter have a 100-MHz bandwidth at a center frequency of 450 MHz (Fig 3). The pulse repetition frequency is 1 MHz and the received pulse is measured using an oscilloscope. As the recommended speed of chest compression is very slow (100 to 120 per minute; < 2 Hz) compared with the IR-UWB signal frequency, we can measure the CCD with a high resolution (< 0.1 mm) through repetitive measurement and averaging. The values of *Δt* are measured using the data from the oscilloscope, and a laptop computer is used to calculate the values of CCD from this data (Fig 1).

### Equipment and protocols

The trials were conducted in the radio frequency and analog circuit design laboratory of the university in August 2016. We used the CPRmeter (Laerdal Medical, Stavanger, Norway) as the accelerometer device and the Resusci Anne QCPR (Laerdal Medical) as the manikin. The CCD values were measured simultaneously using the Resusci Anne QCPR and the CPRmeter or IR-UWB sensor.

The Resusci Anne QCPR could measure the total CCD and leaning depth simultaneously. We calculated the actual CCD by subtracting the leaning depths from the total CCD (Actual CCD = Total CCD—Leaning depths). The actual CCD values obtained using the Resusci Anne QCPR were used as reference CCD values to evaluate the accuracies of the CCD measured by the CPRmeter and the IR-UWB sensor.

For trials 3 and 4, a 4-inch high foam mattress was used. In all the trials, one of the research team member who was a certified Basic & Advanced Life Support instructor by the American Heart Association performed 50 chest compressions according to the feedback signals received from the manikin. The feedback screen of the CPRmeter was blinded, because the chest compressions based on the accelerometer feedback devices could lead to significant under compression in trials 3 and 4 [6]. A 30-min resting period was provided between the trials.

### Statistical analysis

All the statistical analyses were performed using IBM SPSS Statistics, version 23.0 (IBM Corp., Armonk, NY, USA). The variables were expressed as means ± standard deviation. The data were analyzed using Shapiro-Wilk tests to verify the normality of the distribution. For normally distributed data, two-sided student paired *t*-tests were performed; for the remaining data, the Wilcoxon signed rank tests were used to compare the measured CCD obtained from the manikin and the devices (CPRmeter and IR-UWB sensor). In addition, two-sided student *t*-tests or Mann-Whitney *U* tests were used to compare the gaps between the CCD measured by the manikin and the devices on different surfaces. *P* values < 0.05 were considered statistically significant.

## Results

### CCD measured by the Resusci Anne QCPR and the devices (CPRmeter vs. IR-UWB sensor)

**Fig 4:**
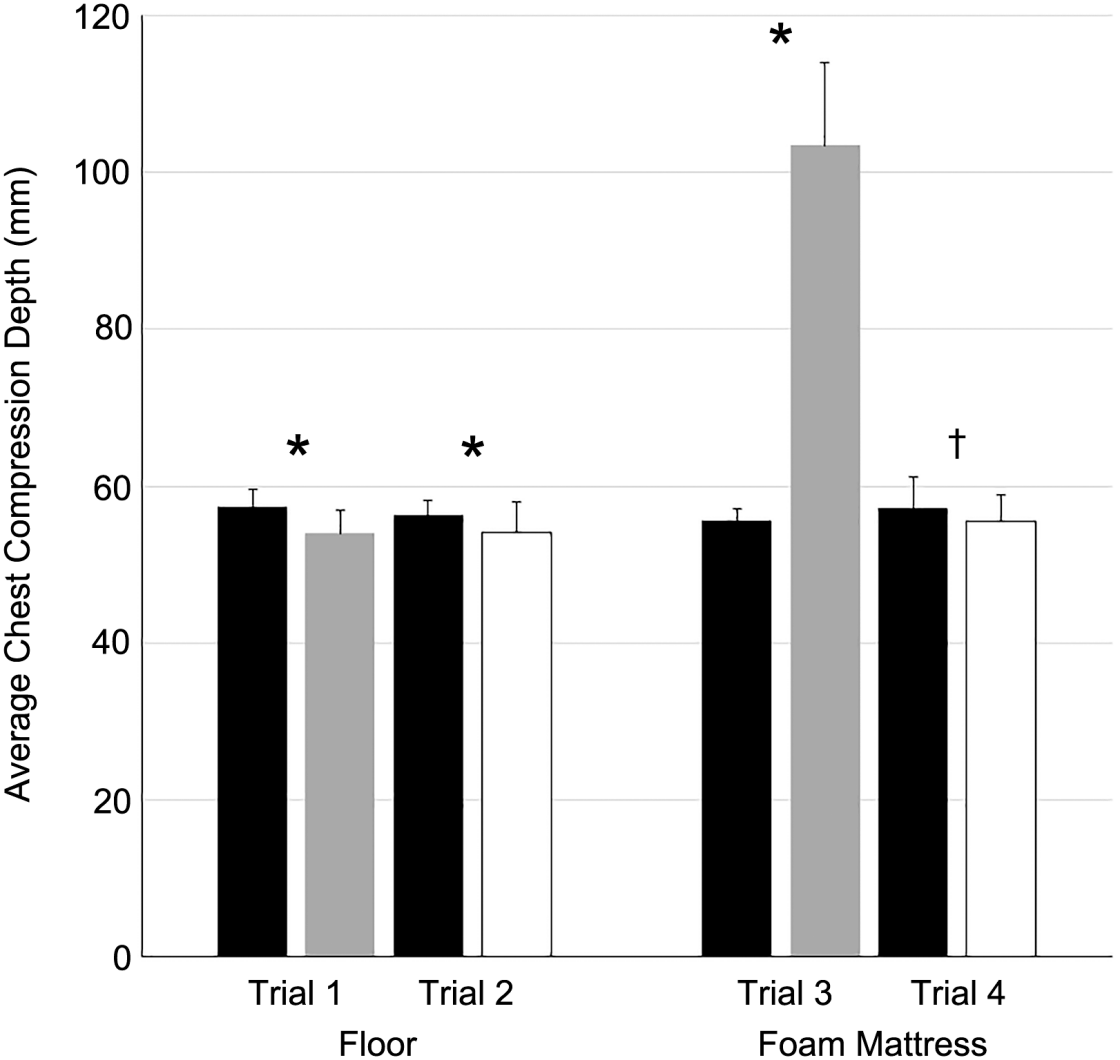
Measured chest compression depths on different surfaces with the CPRmeter (gray bar) and IR-UWB sensor (white bar). The black bars represent the depths measured by the Resusci Anne QCPR. The bars display the means, and the error bars indicate ± SD. * *p*<0.001 Resusci Anne QCPR vs. CPRmeter on all surfaces and Resusci Anne QCPR vs. IR-UWB sensor on the floor; † *p* = 0.012 Resusci Anne QCPR vs. IR-UWB sensor on the foam mattress.

The CCD values measured by the Resusci Anne QCPR and devices are as follows (Fig 4). In trial 1 (Chest compressions on the floor with a CPRmeter), the CCD measured by the manikin and the CPRmeter were 57.42 ± 2.23 mm and 53.92 ± 2.92 mm, respectively (*p* < 0.001). In trial 2 (Chest compressions on the floor with an IR-UWB sensor), the CCD measured by the manikin and the IR-UWB sensor were 56.29 ± 1.96 mm and 54.16 ± 3.90 mm, respectively (*p* < 0.001). In trial 3 (Chest compressions on a foam mattress with a CPRmeter), the CCD measured by the manikin and the CPRmeter were 55.61 ± 1.57 mm and 103.48 ± 10.48 mm, respectively (*p* < 0.001). In trial 4 (Chest compressions on a foam mattress with an IR-UWB sensor), the CCD measured by the manikin and the IR-UWB sensor were 57.14 ± 3.99 mm and 55.51 ± 3.39 mm, respectively (*p* = 0.012).

### Comparisons between the accuracies of the CPRmeter and IR-UWB sensor on different surfaces

The gaps between the CCD measured by the Resusci Anne QCPR and the devices (CPRmeter vs. IR-UWB sensor) on the floor were not much different (3.50 ± 2.08 mm vs. 3.15 ± 2.27 mm, respectively, *p* = 0.136). However, on the foam mattress, the gaps were significantly different (48.53 ± 5.65 mm vs. 4.10 ± 2.47 mm, *p* < 0.001).

## Discussion

The CCD measurements obtained from the accelerometer device were slightly underestimated, compared with those obtained from the manikin [8]. We expected that the CCD measured by the manikin and the accelerometer device would be the same, if the leaning depths were subtracted from the total CCD. However, the accelerometer device still underestimated the CCD slightly on the floor despite subtracting the leaning depths from the total CCD (Fig 4). The IR-UWB sensor also showed a similar pattern, both on the floor and on the foam mattress. In contrast, the accelerometer device overestimated the CCD significantly on the foam mattress, as expected. This result confirms that the IR-UWB could measure the CCD consistently, irrespective of the surface (hard versus soft).

The gap between the CCD value obtained by the manikin and the accelerometer device on a foam mattress in our study was significantly larger than that from the previous studies [6, 8]. This difference might be caused by the differences of the feedback signals. The previous studies relied on the feedback signals from the accelerometer devices [6, 8]. However, the CPR provider in this study performed the chest compressions according to the feedback signals from the manikin. Therefore, all the CCD values measured by the manikin were similar (black bars in the Fig 4). The gap between the CCD values measured by the manikin and the accelerometer device on a foam mattress was significantly larger than the gap between the CCD values measured by the manikin and IR-UWB sensor. This result also supports the view that the IR-UWB can measure the CCD accurately irrespective of the surface.

This study had several limitations. First, although radio-frequency signals can penetrate a human body, the signal propagation speed in human body depends on the permittivity of the human. Therefore, a validation study using a living body will be needed to confirm whether this technology could operate successfully. Second, the IR-UWB sensor used in this study was a developmental prototype. Therefore, it requires a heavy instrumental device such as an oscilloscope to measure the time difference of arrival. Further researches will be needed to develop a commercially available device using this technology. Third, we conducted all trials with manual chest compressions. Although we controlled the qualities of chest compressions similarly by using a feedback signals from the manikin, the qualities of the chest compressions were not same among the trials. Ideally, the chest compression should be conducted by using a calibrated drill press.

## Conclusion

Although the accelerometer feedback device overestimated the CCD on the foam mattress, the IR-UWB sensor could measure the CCD accurately, both on the floor and on the foam mattress.

## Supporting information

S1 Data (XLSX)
